# Incidence of Down Syndrome by maternal age in Chinese population

**DOI:** 10.3389/fgene.2022.980627

**Published:** 2022-08-25

**Authors:** Yi Song, Song Jieping, Zhou Tianshu, Zhang Zhijun, Zhang Jingxuan, Wang Bo

**Affiliations:** ^1^ Medical Genetics Center, Maternal and Child Health Hospital of Hubei Province, Wuhan, China; ^2^ The First Clinical College, Hubei University of Medicine, Shiyan, China; ^3^ Department of Reproductive Medical Center, Taihe Hospital, Hubei University of Medicine, Shiyan, China; ^4^ Hubei Key Laboratory of Embryonic Stem Cell Research, School of Basic Medical Sciences, Hubei University of Medicine, Shiyan, China

**Keywords:** down syndrome, maternal serum screening, advanced maternal age (AMA), Asian population, Hubei China

## Abstract

**Objective:** This study aims to estimate the maternal age-related risk of Down syndrome in an Asian population.

**Methods:** We performed a retrospective data analysis including a total of 206,295 pregnant women who presented for second-trimester maternal serum screening for Down syndrome at Hubei Maternal and Child Health Hospital for the years 2008–2017. Cases were assigned to three groups: ≤26 years of age, 27–33 years of age, and ≥34 years of age. The incidence of Down Syndrome was calculated for each age group. The differences between groups were tested using the chi-square (χ2) test.

**Results:** The incidence of Down syndrome in women ≤26 years of age, 27–33 years of age, and ≥34 years of age was 0.67‰, 0.29‰, and 2.07‰ respectively. Statistically significant difference was found between the three age groups (χ2 = 79.748, *p* < 0.05).

**Conclusion:** Down syndrome rate was significantly higher in women ≥34 years of age. Younger women (≤26 years of age) had a significantly higher risk for Down’s syndrome, compared to women aged 27–33.

## Introduction

Down Syndrome (DS) is the most commonly recognized genetic cause of intellectual disability, which occurs in 3.05 per 10,000 live births in China (Deng et al., 2015; Zhang et al., 2020). Since there is no medical cure for DS, it imposes an enormous financial burden on affected families and the health care system (Chen et al., 2008). The average lifetime economic burden of a new DS case from the family perspective and the societal perspective amounted to US$47,000 and US$55,000, respectively (Luo et al., 2020). People have recognized Down Syndrome, although prenatal screening tests are not mandatory in China, most pregnant women had this test during pregnancy in China.

Advanced maternal age (AMA) is a well-established risk factor for DS and an essential determinant in all prenatal screening strategies (Resta, 2005; Bhaumik et al., 2017; Chen et al., 2020). The association between maternal age distribution and the live birth prevalence of DS has been well documented in American and Europe (McKenzie et al., 2016). However, racial-ethnic differences exist in prenatal diagnostic test use and associated outcomes of AMA (Khoshnood et al., 2000; Khattak et al., 2019). In this study, we estimated the maternal age-related risk of Down syndrome in an Asian population.

## Materials and methods

### Subjects

We performed a retrospective data analysis including a total of 206,295 pregnant women who presented for second-trimester maternal serum screening for Down syndrome at Hubei Maternal and Child Health Hospital for the years 2008–2017.

### Maternal serum screening

For each pregnant woman, 2 ml of maternal peripheral blood was collected and conserved at 4°C after separation of serum. The serum biomarkers, AFP, μE3, and β-HCG were detected by chemiluminescent immunoassay. The TCsoft prenatal screening software was used to calculate the screening risk. Risk = Age-specific Risk*LR (AFP)*LR (β-HCG) *LR (μE3). The initial MOM value was corrected by body weight, race, multiple births, diabetes, and other factors. The cut-off value for DS risk was set at 1:380.

### Down syndrome diagnosis

Before 2014, pregnant women with a positive screening result were diagnosed with amniocentesis. After 2014, pregnant women with a positive screening result were diagnosed by amniocentesis or screened by non-invasive prenatal screening (NIPS).

### Statistical analysis

All cases were assigned to three groups: ≤26 years of age, 27–33 years of age, and ≥34 years of age. The incidence of Down’s Syndrome was calculated for each age group. The differences between groups were tested using the χ2 test. All the analyses were performed by SPSS 16.0 for Windows (SPSS Inc. Chicago, IL, United StatesUnited States). A *p* value of <0.05 was considered statistically significant.

## Results

### The incidence of DS by maternal age

The incidence of DS by maternal age was summarized in Table 1. In a total of 206,295 cases, 13,100 (6.35%) had positive screening tests (DS risk > or = 1:380). 93 (89.4%) fetuses were diagnosed with DS prenatally and 11 (10.6%) fetuses were diagnosed postnatally (these 11 newborns have been genetically confirmed with full/partial trisomy 21 after birth), 78.85% (82/104) of children with Down syndrome are born to women under 35 years of age. (Table 1).

**TABLE 1:** Incidence of DS by maternal age.

Maternal age	Diagnosed cases			Total cases	Incidence (‰)
	Diagnosed antenatally	Diagnosed postnatally	Total		
18–21	1	0	1	3,061	0.33
22	2	0	2	3,294	0.61
23	5	0	5	5,985	0.84
24	3	2	5	9,876	0.51
25	8	3	11	14,560	0.76
26	12	2	14	19,955	0.70
27	6	1	7	26,409	0.27
28	10	1	11	28,267	0.39
29	6	1	7	26,517	0.26
30	2	0	2	20,152	0.10
31	6	0	6	14,753	0.41
32	2	0	2	11,259	0.18
33	4	0	4	9,194	0.44
34	5	0	5	6,726	0.74
35	4	0	4	3,602	1.11
36	3	1	4	1,203	3.33
37	4	0	4	623	6.42
38	1	0	1	364	2.75
39	1	0	1	203	4.93
≥40	8	0	8	292	27.40
Total	93	11	104	206,295	0.50

### Incidence of Down syndrome by maternal age groups

All cases were assigned to three groups: ≤26 years of age, 27–33 years of age, and ≥34 years of age. The incidence of DS in women ≤26-years of age, 27–33 years of age, and ≥34 years of age was 0.67‰ (38/56,731), 0.29‰ (39/136,551), and 2.07‰ (27/13,013) respectively (Table 2). Statistically significant difference was found between the three age groups (χ2 = 79.748, *p* < 0.05) (Table 2). Remarkably, the proportion of pregnant women ≤26-years of age was 27.5% (56,731/206,295) and the incidence of DS in this age group is significantly higher than women 27–33 years of age (Table 2).

**TABLE 2:** Incidence of DS by maternal age groups.

Maternal age	Total cases	Diagnosed cases	Incidence (‰)^a^
≤26	56,731	38	0.67
27–33	136,551	39	0.29
≥34	13,013	27	2.07

a The incidence of DS, was significantly different among the groups (χ2 = 79.748, *p* < 0.05).

## Discussion

Down syndrome (DS) can occur at any maternal age but the chance of having a child with DS increases over time. It has been well accepted that women over age 35 at delivery are at higher risk for giving birth to a child with DS. In fact, for a long time, advanced maternal age (AMA) has been considered as a sole indication for genetic amniocentesis for definitive prenatal diagnosis (Bornstein et al., 2009; Stomornjak-Vukadin et al., 2015). In our study, the incidence of DS in women over age 34 was significantly higher than in other age groups (Table 2). These data are consistent with previous research (Howe et al., 2000; Morris et al., 2002). Fortunately, women in this age group are usually well informed about the age-associated risk and treated as if they need the level of care necessary for any high-risk pregnancy. Our data showed all diagnoses in this age group were made prenatally except one (Table 1).

A trend has developed worldwide for women to delay childbearing into their late 30 s or early 40 s. In China, at end of October 2015, the one-child policy was replaced by a universal two-child policy. As a result, the proportion of women with AMA at delivery increased by 85.68%, from 8.52% in 2013 to 15.82% in 2017 in Zhejiang province (Zhang et al., 2020). The results of our study, which analyze a population of pregnant women in Hubei province, showed the proportion of women over age 34 was only 6.3% between 2008 and 2017 suggesting that there are considerable variations in the prevalence of AMA across the county (Table 2). Future studies are required to confirm the trend and understand the demographic differences.

Previous studies conclude there is no association between younger maternal age and the risk of DS. However, we found that women under age 26 (18–26) account for nearly 27% of all pregnant women and are more likely to have babies with DS than women aged 26–34 (χ2-14.858 *p* < 0.01) (Table 2). This is partly due to the increasing availability of more powerful tests, which significantly increased the overall detection rate. Another explanation could be that young mothers lack all awareness of pregnancy and therefore are more likely to engage in risk behaviors such as smoking, drinking, and using illicit drugs that can induce chromosomal non-disjunction (Czeizel, 1990; Sotonica et al., 2016). They are also more likely to be sleep-deprived, have an imbalanced diet to control body weight. Recent studies have identified some genetic predispositions (just as consanguineous marriage, maternal telomere length, maternal MCM9 polymorphisms, maternal Presenilin-1 and Apolipoprotein E polymorphisms) of women that may cause nondisjunction and Down syndrome birth at younger age (Ray et al., 2016; Bhaumik et al., 2017; Ray et al., 2018; Pal et al., 2021). These are age-independent risk factors. Epidemiological risk factors are not only responsible for DS birth to younger mother. In our study, 18.4% (7/38) of DS in women ≤26 years of age are diagnosed postnatally, compared to 7.7 (3/39) in the women aged 26–34 and 3.7% (1/27) in the woman ≥34 years of age (Tables 1,2). In an early study, it predicts that with continued use of prenatal diagnosis among older women, upward of 80% of DS births will occur to younger mothers (Adams et al., 1981; Antonarakis et al., 2020). Our study shows high occurrence of DS in women younger than 26 years. Therefore, much more attention needs to be given to younger mothers. Maternal serum screening for DS and/or NIPT should be routinely performed to all pregnant women. Healthcare professionals need to help them to understand the importance of this screening test and make informed decisions.

## Conclusion

The incidence of DS was significantly higher in women ≥34 years of age. Younger women (≤26 years of age) had a significantly higher risk for DS, compared to women aged 27–33. The strengths that Younger women (≤26 years of age) had a significantly higher risk for DS, compared to women aged 27–33. This could help to increase the awareness in these young mothers to prevent the occurrence in other young mothers. Much more attention needs to be given to younger mothers. Maternal serum screening for DS and/or NIPT should be routinely performed to all pregnant women.

## Data Availability

The original contributions presented in the study are included in the article/supplementary materials, further inquiries can be directed to the corresponding authors.
